# Effect of late-gestation heat stress in nulliparous heifers on postnatal growth, passive transfer of immunoglobulin G, and thermoregulation of their calves

**DOI:** 10.3168/jdsc.2020-0069

**Published:** 2021-03-12

**Authors:** B.D. Davidson, B. Dado-Senn, V. Ouellet, G.E. Dahl, J. Laporta

**Affiliations:** 1 Department of Animal Sciences, University of Florida, Gainesville 32608; 2 Department of Animal and Dairy Sciences, University of Wisconsin, Madison 53706; 3 Department of Animal Sciences, Université Laval, Québec City, Québec, Canada G1V 0A6

## Abstract

Youngstock such as nulliparous pregnant dairy heifers are not typically considered for active cooling, as they are thought to be more heat tolerant than mature cows. Recently, the benefits of heat stress abatement in pregnant heifers were studied, but the effect of in utero hyperthermia on the calf is still unknown. Herein, we aimed to investigate the effects of late-gestation heat stress in nulliparous heifers on the growth, immune, and thermoregulatory responses of their calves. Pregnant nulliparous dams were randomly selected for either active cooling (CL; fans and soakers; n = 15) or heat stress (HT; n = 16) 60 d before expected calving. After birth, respiration rate, rectal temperature, skin temperature, and sweating rate were obtained from their heifer calves at 24 h and on d 14, 28, 42, and 56. Blood samples and body weights were both collected for measurement of total protein and hematocrit and calculation of average daily gains, respectively. Gestation length was shorter in HT heifers (272 vs. 276 ± 1.28 d) relative to CL heifers. Birth weights, weaning weights, body weights up to d 56, and average daily gain from birth to weaning were not different between in utero HT (IUHT; n = 13) and in utero CL (IUCL; n = 12) calves. Apparent efficiency of absorption of immunoglobulin G tended to be lower in IUHT calves (26.3 vs. 42.7 ± 9.0%), and serum IgG concentrations from birth to d 56 were significantly lower in IUHT calves relative to IUCL calves (22.0 vs. 32.4 ± 4.47 g/L). Postnatal respiration rate, rectal temperature, sweating rate, total protein, and hematocrit were not different between in utero treatments. There was a tendency for IUHT calves to have lower skin temperature at 24 h (34.9 vs. 36.9 ± 1.05°C), and skin temperature tended to be elevated in IUHT calves at d 56 (29.6 vs. 27.9 ± 1.05°C). In summary, the lack of heat stress abatement during late gestation reduces gestation length of nulliparous heifers. Additionally, providing active cooling to nulliparous heifers during the prepartum period confers immune benefits to their offspring, although it does not translate to growth improvements.

## Highlights

• Late-gestational heat stress in nulliparous heifers affects the in utero calf postnatally.
• Apparent efficiency of IgG absorption at birth and serum IgG concentrations throughout the preweaning period are lower in offspring born to heat-stressed heifers.
• Postnatal birth weights, growth trajectories, blood total protein, and hematocrit are not affected by the prenatal intrauterine environment.
• Postnatal thermoregulation measures varied slightly as an effect from the intrauterine environment.

## Graphical Abstract

**Figure:**
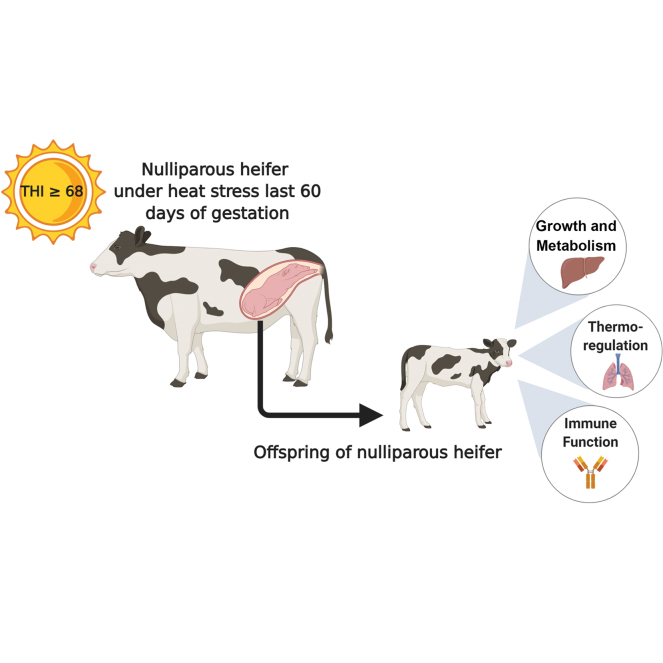
**Summary:** Pregnant, nulliparous heifers that were heat stressed during late gestation produced heifer calves that were negatively affected in terms of growth and metabolism, immune function, and thermoregulation. These in utero heat-stressed heifer calves suffered negative outcomes during the postnatal period, which was evidenced by decreased apparent efficiency of absorption of immunoglobulin G after colostrum feeding, decreased serum immunoglobulin G concentrations throughout the preweaning period, and increased surface temperatures at weaning.

Heat stress exposure during the dry period affects not only the multiparous pregnant cow but also the fetus in utero, as it coincides with late gestation. Adverse effects of late-gestation heat stress on the calf have been extensively documented and include compromised fetal growth and development, decreased gestation length (Tao et al., 2012; Monteiro et al., 2014), and lower birth weight (Collier et al., 1982; Tao et al., 2012; Dado-Senn et al., 2020b). Additionally, Tao et al. (2012) reported that in utero heat-stressed (**IUHT**) calves had decreased passive immune transfer and cellular immune function as well as reduced plasma total protein and hematocrit (**HCT**) compared with in utero cooled (**IUCL**) calves. It was recently established that nulliparous, pregnant heifers are also negatively affected by heat stress, as evidenced by elevated thermoregulatory responses and reduced milk production in their first lactation (Davidson et al., 2021). Notably, the magnitude of differences in the thermoregulatory responses and production variables between cooled and heat-stressed pregnant heifers was not as large as observed differences in multiparous lactating or dry pregnant cows. To date, it is unknown whether the intrauterine environment of actively cooled and heat-stressed nulliparous pregnant heifers exerts similar or different outcomes on the postnatal life of the offspring. Herein, we investigated the effects of late-gestation heat stress in nulliparous heifers on the thermoregulatory, growth, and immune responses of their postnatal calves. We hypothesized that IUHT calves born to nulliparous heifers would be born lighter, have impaired growth, and thermoregulate suboptimally postnatally compared with their IUCL counterparts.

This experiment was approved by the Institutional Animal Care and Use Committee at the University of Florida and was conducted at the University of Florida Dairy Unit (Hague) from June to November 2019. Thirty-one nulliparous pregnant Holstein heifers were randomly assigned to a heat stress (**HT**; n = 16) or cooling (**CL**; n = 15) treatment 60 d (average treatment duration: 55 ± 5 d) before expected calving date, blocked by BCS and genomic PTA for milk (Enlight and Clarifide, Zoetis Services LLC). All heifers were housed in the same sand-bedded freestall barn, where the HT pens were provided only with shade of the open-sided barn and the CL pens were provided with shade of the barn, water soakers over the feed line, and fans over the stalls. Details on maternal treatments, thermoregulatory measures, and diets were previously reported by Davidson et al. (2021).

Heifer calves, either IUHT (n = 13) or IUCL (n = 12), born to these nulliparous heifers were followed from birth through complete weaning (56 d of age). The calves were housed in individual pens inside an open-sided barn equipped with overhead fans and raised according to the standard operating procedures of the University of Florida Dairy Unit. Within 4 h of birth, calves were separated from their dams, weighed, and fed 3.7 L of thawed high-quality colostrum (i.e., ≥22% Brix refractometer reading) in a single feeding. Colostrum did not come from their respective dam. Samples of fed colostrum (n = 9–10/treatment; 10 mL) were collected and stored at −20°C for IgG analysis. Daily feedings included 3.7 L of pasteurized waste milk 2× daily at 0700 and 1500 h until 49 d, when milk weaning began and calves were fed 3.7 L 1× daily at only 0700 h. Complete milk weaning occurred on 56 d. Water was provided ad libitum, and water intake was not recorded. At 1 d of age (28.8 ± 7 h after birth), blood was collected and respiration rate (**RR**), rectal temperature (**RT**), skin temperature (**ST**), and sweating rate (**SR**) were recorded. These measurements were repeated at d 14, 28, 42, and 56 between 1300 and 1500 h. Beginning at 3 d, starter grain (Ampli-Calf Starter 20 Warm Weather; Purina Animal Nutrition LLC) was provided ad libitum, and intakes were not recorded.

Blood samples were collected by jugular venipuncture using 2 Vacutainer tubes (10 mL, Becton Dickinson), one containing clot activator gel (cat. no. 366430, Becton Dickinson; placed at room temperature for approximately 30 min) and one containing sodium-heparin anticoagulant (cat. no. 366480, Becton Dickinson; placed on ice). Hematocrit was assessed from whole blood from the sodium-heparinized tube. Capillary tubes (no. 22-362-566, Fisher Scientific) filled with whole blood were centrifuged at 2,240 × *g* for 3 min at room temperature in a microhematocrit centrifuge and read with a circular microcapillary HCT reader. Then, serum and plasma were separated through centrifugation at 3,000 × *g* for 20 min at room temperature. Serum was aliquoted and stored at −20°C. Total protein was assessed from plasma using a digital Brix refractometer (MA871, Milwaukee Instruments) before it was aliquoted and stored at −20°C.

From thawed serum and colostrum samples, IgG concentrations were calculated to quantify apparent efficiency of absorption and serum IgG concentrations from birth to weaning. Apparent efficiency of IgG absorption (%) was estimated indirectly by measuring the IgG content in colostrum fed to the calf and serum at 24 h after colostrum feeding using the single radial immunodiffusion test (Bovine IgG Test Kit; Triple J Farms) according to the manufacturer's protocol. Calculations were performed according to the equation from Quigley and Drewry (1998): serum IgG (g/L) × birth weight (kg) × serum volume (%)/IgG fed (g) × 100. From biweekly BW, ADG was calculated by subtracting 24-h weights from d 56 weights and dividing by 56. Respiration rate was measured by counting flank movements for 1 min (breaths/min), RT was measured using a digital thermometer (Sharptemp V Large Animal Digital Thermometer, PBS Animal Health), ST was obtained with an infrared thermometer (Raytek MiniTemp MT6 Infrared Thermometer; Instrumart), and SR was measured with a VapoMeter (cat. no. SWL5042, Delfin Technologies).

All statistical analyses were performed in SAS (version 9.4, SAS Institute Inc.). Data were tested for covariance (Levene's test), and normality was tested by evaluating the Shapiro-Wilk statistic using the Univariate procedure. Using a generalized linear mixed model, gestation length, ADG, birth weight, weaning weight, and apparent efficiency of absorption were analyzed. Models included the main effect of treatment and calf ID nested within treatment as the random effect. All other variables were analyzed using the Mixed procedure. The model included fixed effects of treatment, day, and their interaction, and animal ID nested within treatment was used as the random effect. Significance was declared at *P* ≤ 0.05, and tendency was declared at 0.05 < *P* ≤ 0.10. Data are presented as least squares means ± standard error unless otherwise stated.

Throughout the duration of the prenatal experiment, temperature-humidity index (**THI**) of the experimental pens averaged 77.33 ± 0.20, calculated according to the equation recommended by Dikmen and Hansen (2009): THI = (1.8 × T + 32) − [(0.55 − 0.0055 × RH) × (1.8 × T − 26)], where T = ambient temperature (°C) and RH = relative humidity (%). Briefly, from Davidson et al. (2021), nulliparous pregnant heifers with access to active cooling during the last 8 wk of gestation had lower RR (44.3 vs. 60.0 ± 1.6 breaths/min, *P* < 0.0001), RT (38.7 vs. 38.8 ± 0.04°C, *P* = 0.007), ST (34.7 vs. 35.3 ± 0.17°C, *P* = 0.002), and SR (19.0 vs. 35.2 ± 1.90 g/m^2^ per hour, *P* < 0.0001) relative to the HT heifers. During the precalving period, DMI was not different (7.99 vs. 8.34 ± 1.03 kg/d, CL vs. HT, *P* = 0.76) between treatments, and postcalving milk production was higher in CL dams (35.8 vs. 31.9 ± 1.4 kg/d, CL vs. HT, *P* = 0.01) than in HT dams. Gestation length was shorter in the HT heifers (*P* = 0.003; Table 1).

**Table 1:** Gestation length (GL), birth weight, weaning weight, ADG, BW, total protein (TP), and hematocrit (HCT) from heifer calves gestated by nulliparous heifer dams that were exposed to either cooling or heat stress environments during the last 60 d of gestation

Variable	Treatment^1^		SEM	*P*-value		
	Cooled	Heat stressed		Treatment	Day	Treatment × day
GL, d	276.4	272.1	1.28	0.003	—	—
Birth weight, kg	34.89	34.33	1.32	0.68	—	—
Weaning weight, kg	68.16	66.79	1.95	0.49	—	—
ADG,^2^ kg/d	0.60	0.57	0.03	0.39	—	—
BW,^3^ kg	50.99	49.43	1.39	0.27	<0.0001	0.99
TP, mg/dL	8.40	8.28	0.18	0.50	<0.0001	0.81
HCT, %	31.01	30.66	0.93	0.71	0.0004	0.56

1 Heifer dams were cooled (shade of barn, fans, soakers; n = 15) or heat stressed (shade of barn; n = 16) during the last 60 d of gestation.

2 Average daily gain was calculated by subtracting calf BW at birth from BW at d 56 (weaning), divided by 56.

3 Body weights were measured at 24 h (d 1) and at d 14, 28, 42, and 56.

Calf birth weights, biweekly calf BW, and weaning weights were not different between treatments (*P* > 0.27; Table 1), and ADG from birth to weaning did not differ between treatments (*P* = 0.39; Table 1). Concentration of IgG in colostrum samples fed to IUCL (n = 9) and IUHT (n = 10) calves was similar (109.9 vs. 116.3 ± 14.2 g/L, respectively; *P* = 0.65); however, the apparent efficiency of absorption tended to be lower in the IUHT calves compared with the IUCL calves (26.3 vs. 42.7 ± 9.01%, *P* = 0.09; Figure 1A). Through the preweaning period, serum IgG concentrations were reduced in the IUHT calves relative to the IUCL calves (22.0 vs. 32.4 ± 4.47 g/L, *P* = 0.03; Figure 1B). An effect of day (*P* < 0.0001) was detected, whereby serum IgG decreased from birth to d 42 and increased between d 42 and d 56. Total protein and blood HCT were not different between treatments (*P* ≥ 0.50; Table 1), but there was an effect of day (*P* < 0.0004). The interaction between in utero treatments and days was not different for both total protein and blood HCT (*P* > 0.56).

**Figure 1:**
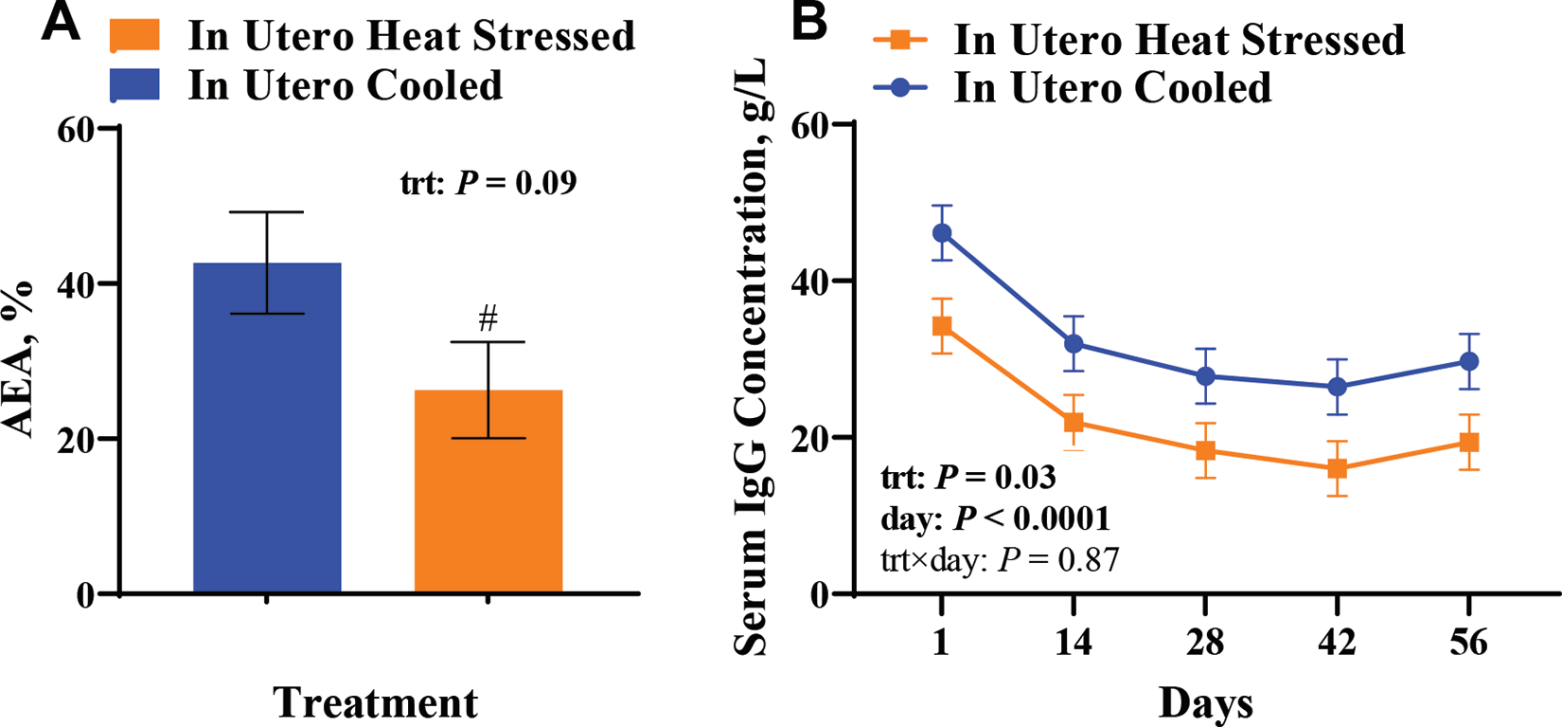
Apparent efficiency of absorption (A) and concentrations of serum IgG (B) from heifer calves born to nulliparous heifer dams that were cooled (shade of a freestall barn, fans, and soakers; n = 9) or heat stressed (shade of a freestall barn; n = 10) during the last 60 d of gestation. Blue bar and line indicate in utero cooled calves, and orange line and bar indicate in utero heat-stressed calves. Apparent efficiency of absorption (AEA) was calculated with estimates and the formula outlined by Quigley and Drewry (1998) as follows: AEA (%) = [plasma IgG (g/L) × birth weight (kg) × plasma volume (L)/IgG fed (g) × 100]. trt = treatment. Symbol (#) indicates that means differ (0.10 ≥ *P* > 0.05). Data are LSM ± SE.

The RR, RT, and SR were not different between in utero treatments (all *P* ≥ 0.28; Figure 2), but RR, RT, and ST had day effects (*P* ≤ 0.0007). Respiration rates (Figure 2A) decreased from birth until d 56, and RT (Figure 2B) increased from 24 h to d 14, decreased from d 14 to d 28, and increased from d 28 to d 56. There was a tendency (*P* = 0.08; Figure 2D) for a treatment × day interaction for ST, whereby ST tended to be lower in the IUHT calves at 24 h (34.9 vs. 36.9 ± 1.05°C, *P* = 0.06) and tended to be higher in the IUHT calves at d 56 (29.6 vs. 27.9 ± 1.05°C, *P* = 0.10) relative to the IUCL calves.

**Figure 2:**
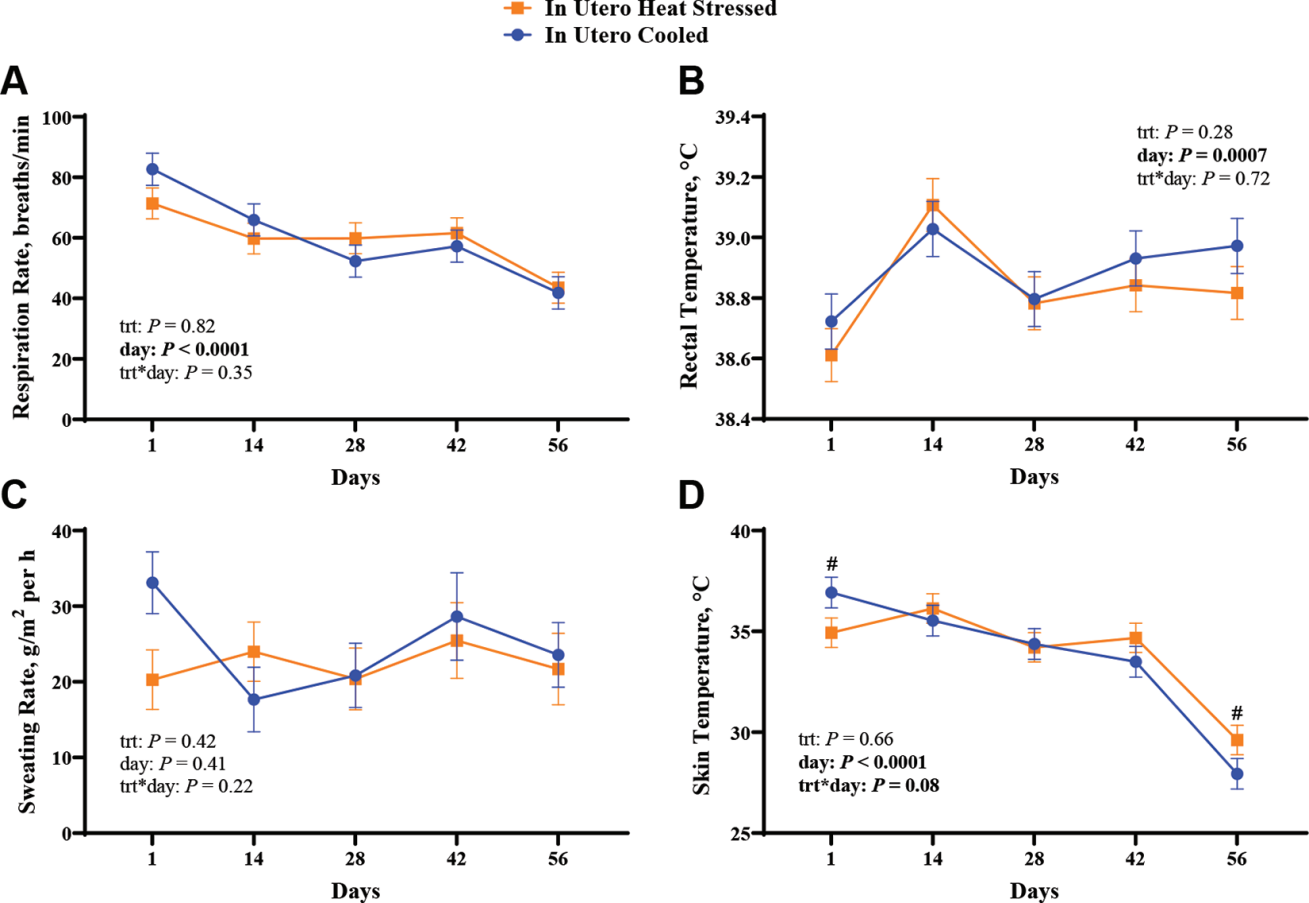
Respiration rate (A), rectal temperature (B), sweating rate (C), and skin temperature (D) measurements from heifer calves born to nulliparous heifer dams that were cooled (shade of a freestall barn, fans, and soakers; n = 12) or heat stressed (shade of a freestall barn; n = 13) during the last 60 d of gestation. Blue circle and line indicate in utero cooled calves, and orange square and line indicate in utero heat-stressed calves. Relative to calving, measurements were obtained at 24 h (d 1) and at d 14, 28, 42, and 56. trt = treatment. Symbol (#) indicates that means differ (0.10 ≥ *P* > 0.05). Data are LSM ± SE of the interaction (treatment × time).

Heat stress in mature lactating and dry dairy cows has been studied extensively; however, the effect of heat stress on nulliparous pregnant dairy heifers and their calves has received less attention. Herein, we investigated the effects of in utero heat stress on the growth, passive immunity, and thermoregulatory responses of calves born to nulliparous dams. Although all dams were exposed to the same elevated THI during late gestation, RR, RT, ST, and SR were reduced in CL heifers relative to HT heifers. This suggests that the CL animals experienced reduced heat strain and that we successfully achieved different treatments to effectively study heat stress during the late-gestational period in heifers and the effects on their offspring.

In the present study, no differences were observed in calf birth weight, weaning weight, BW up to d 56, or ADG during the preweaning period. Conversely, calves born to multiparous cows cooled during the dry period are heavier at birth (Collier et al., 1982; Monteiro et al., 2014, 2016b; Tao et al., 2014) and gain more per day during the preweaning period (Laporta et al., 2017) relative to calves born to multiparous cows exposed to heat stress during the dry period. However, even though reduced offspring birth weights are a benchmark of in utero heat stress exposure in cows and other species, the lack of difference in BW gains from birth to weaning agrees with other studies using multiparous cows in a similar model (Tao et al., 2012; Monteiro et al., 2016b). Those authors suggest that the lack of difference in calf weight at weaning is a consequence of different birth weights and not altered growth rates (Tao et al., 2012; Monteiro et al., 2016b). In the present study, there were neither birth weight nor weight gain differences, which supports the premise that nulliparous heifers and their calves' growth are not affected to the same degree by heat stress as mature dry cows.

Notably, in multiparous cow and calf pairs, reduced birth weight upon in utero heat stress exposure is typically attributed to a combination of decreased gestation length and compromised fetal growth, likely due to impaired placental function (do Amaral et al., 2009; Monteiro et al., 2014; Dado-Senn et al., 2020a). Although the reduction in gestation length in HT nulliparous heifers was of similar magnitude to that observed in multiparous cows (4 d vs. 3.2 d, heifer dams vs. multiparous dams, respectively; reviewed by Ouellet et al., 2020), the typical calf birth weight reduction due to multiparous dam hyperthermia was not observed in the offspring of nulliparous heifers. Altogether, this suggests a possible higher heat stress tolerance of primiparous pregnant dams and the developing fetus and that the IUHT calves might have a higher fetal growth rate. Monteiro et al. (2016a) reported that maternal exposure to heat stress in multiparous cows affects their respective calves' HCT, whereby IUCL calves tended to have higher HCT levels compared with their IUHT counterparts. In the ovine model, 2- to 3-yr-old pregnant ewes exposed to late-gestation heat stress displayed intrauterine hypoxia due to impaired placental function (Regnault et al., 2007). It is suggested that a similar hypoxia and ensuing carryover effect occur in the bovine, whereby IUHT calves from multiparous dams adapt to decreased amounts of uterine oxygen, leading to decreased HCT levels across the preweaning period (Monteiro et al., 2016a). However, in the present study no differences were found in HCT for the calves born to heifer dams. This further supports the postulation that heifer dams and their calves are more heat tolerant, potentially as a result of a milder effect of heat stress on placental function during late gestation. However, this postulation warrants further investigation.

Although concentrations of IgG in colostrum samples fed to the calves were similar between treatments, apparent efficiency of absorption and serum IgG concentrations were elevated in prenatally CL calves, which agrees with findings from multiparous dams (Tao et al., 2012; Monteiro et al., 2014; Laporta et al., 2017). Impairment of passive transfer in prenatally heat-stressed calves may be attributed to reduced postnatal absorption in the small intestine, as a result of impaired intestinal development and decreased surface area for absorption (Ahmed et al., 2016; reviewed by Dado-Senn et al., 2020a). The present results suggest that calves gestated by nulliparous heifers and exposed to in utero heat stress might suffer from reduced small intestine development and function to a greater degree than any other growth parameter.

Last, no differences were observed between treatments in calf SR, RT, or RR in the present study, but there was a tendency for ST to be lower at 24 h and higher at d 56 in IUHT calves compared with IUCL calves. These findings are not in accordance with calves born from multiparous cows, where IUHT calves have higher RT relative to IUCL calves (Laporta et al., 2017). The cause of altered ST is unknown but suggests an enhanced ability of IUHT calves to thermoregulate immediately after parturition. However, by weaning age, IUCL calves have lower ST and demonstrate an advanced ability to thermoregulate, potentially granted to them from their actively cooled in utero environments. These postulations warrant further molecular investigation.

In conclusion, calves born to heat-stressed primiparous heifers suffered negative outcomes that were evident during the postnatal period, though the effect was notably less pronounced relative to calves born to multiparous heat-stressed cows. Regardless, calves born to heat-stressed primiparous heifer dams exhibited increased ST at weaning, were less efficient at absorbing immunoglobulins from colostrum, and had impaired levels of serum IgG through the preweaning period, which suggest a reduced capacity to respond to health challenges in early life.
